# Oro-Motor Intervention Protocol to Improve Sucking Behavior among Neonates with Immature Sucking: An Experimental Protocol

**DOI:** 10.29337/ijsp.152

**Published:** 2021-07-23

**Authors:** Ramya Chandran, Jagatheesan Alagesan

**Affiliations:** 1 Saveetha College of physiotherapy, Saveetha Institute of Medical and Technical Sciences, Chennai, Tamilnadu, India

**Keywords:** sucking behavior, pacifier, infant nutrition disorders, newborn

## Abstract

**Background:**

Oro-motor intervention methods were previously adopted to improve the sucking pattern but there is still a lag in the structured protocol for improving sucking behavior in infants with immature sucking. Thus, this study is aimed to develop a structured protocol for the Oro-motor intervention to improve sucking behavior.

**Method:**

Using the prospective observational study design, neonates with poor suck (producing less than 10 sucks per minute), under NG tube feeding, and maintaining oxygen saturation at room air were included. A total of 6 subjects were enrolled in this study and they were treated with Oro-motor intervention protocol. The Sucking rate and LATCH score were taken as the outcome measures and measured at beginning of intervention and after 2 weeks of intervention.

**Result:**

The mean pre-test and post-test values for sucking rate were is (8.66), (32.5) and LATCH were (4.66), (8.16) respectively. The data collected showed that the protocol framed for Oro-motor intervention was significantly effective in improving quality of feeding among infants with immature sucking behavior.

**Conclusion:**

The structured Oro-motor intervention protocol improves the feeding performance in infants with poor sucking behavior and improves the LATCH score. All the infants included in this study where under nasogastric tube feeding, thus the structured protocol can be considered to be helpful in weaning from NG tube feeding.

## Introduction

Effective direct breastfeeding provides short and long duration benefit to mother as well as infant [1]. Direct breastfeeding is the natural method of infant feeding through the mother’s nipple to infant’s mouth which is needed for first six to seven months of infant life. Direct breastfeeding not only fulfills the nutritional status of the infants but also necessary to gain adequate immunity against infections such as meningitis, pneumonia, diarrhea, and reduces mortality rate in infant [2,3]. Direct breastfeeding helps for appropriate weight gain in neonates and reduces length of stay in the hospital [4].

Sucking pattern usually composed of synchronized rhythmic suck, swallow and breathing. It contains alternate suck and expression [5,6]. Nutritive and non-nutritive are the two variations in sucking [7,8]. Primitive reflex sucking develops at 28 weeks of gestation, preterm infants might have immature primitive sucking reflex and leads to poor weight gain during the neonatal period [9,10]. Major cause for poor sucking includes preterm birth, neonates with low birth weight, cleft lip, cleft palate and some other congenital abnormalities [11,12,13]. Term neonates also have poor sucking because of mother experiencing medical complication during antenatal period, intra ventricular hemorrhage and due to type of delivery. Poor sucking behavior refers to the reduced milk intake capacity of the infant resulting in malnutrition. The LATCH scale was designed to quantitatively measure the feeding capability of both mother and the infant, thus reduced latch scores were considered to be a major sign of poor sucking behavior leading to complications such as high risk of aspiration and infections like pneumonia [14]. Neonates with feeding difficulty may be in nasogastric tube to avoid such incidence. Traditional methods help in improving feeding pattern in neonates [15,16].

Sucking pattern was observed during direct breast feeding, the movement of the jaw was noted and it was considered as 1 suck during the observation process. Various treatment methods were previously adopted to improve the sucking pattern, but there is still a lag in the structured protocol for improving sucking behavior in infants with immature sucking [17,18,19]. Thus, the research question of this study was to determine whether there is any significant effect of structured Oro-motor intervention protocol to improve sucking behavior among infants with poor sucking behavior, with a hypothesis that the Oro-motor intervention will be significantly effective in improving sucking behavior.

## Method

### Study design

This was a prospective observational study.

### Sample type

Purposive sampling was done.

### Study setting and duration of the study

Study was conducted at the physiotherapy department of Saveetha medical college and hospital (SMCH) in collaboration with the Neonatal intensive care unit (NICU) of the Pediatrics department, SMCH, Chennai, Tamilnadu, India during the period between the beginning of January 2021 and the end of March 2021.

### Ethical and consent

This Prospective observation study was approved from Institutional Scientific Review Board, in saveetha institute of medical and technical science (27/03/2020/ISRB/MPT/SCPT). Informed consent in written format was obtained from the parents of neonates enrolled in this study after explaining about the study procedure.

### Selection criteria

**Figure 1:**
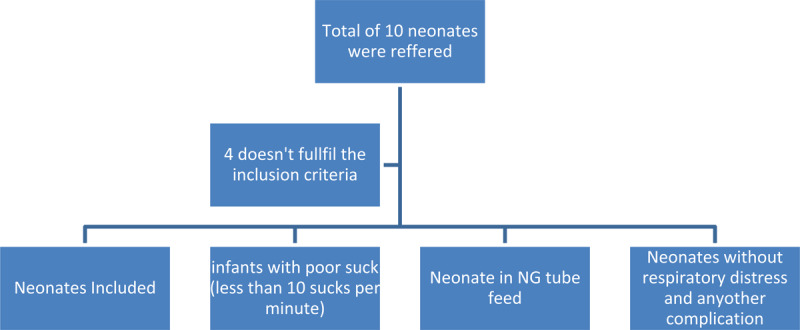
Flowchart representing the selection process.

All preterm and term neonates with poor sucking were included in this study through referral from the neonatologists. The inclusion criteria were to include neonates having poor suck (producing less than 10 sucks per minute), Neonates feeding through NG tube, without respiratory distress and maintaining oxygen saturation at room air. Whereas, neonates with respiratory complications, intraventricular hemorrhage, neonates under mechanical ventilator, congenital malformations such as cleft lip or cleft palate, and mothers with breast abnormalities were excluded the flowchart representing the selection process is shown in ***Figure 1***.

## Data collection

A total of 6 subjects were enrolled in this study and they were treated with Oro-motor intervention protocol. Outcome measures were sucking rate, and LATCH score. Each subject’s sucking rate was recorded by two methods. Asking number of sucks felt by the mother per minute using stopwatch and another through gloved finger by the therapist per minute using stopwatch.

Sucking rate per minute was calculated before the intervention. Number of sucks felt by the mother was calculated during direct breast-feeding after the intervention. Sucking rate was measured before the intervention daily, sucking rate was measured using a stopwatch for continuous 3 minutes and the average value of 2nd minute was taken into consideration [20].

The LATCH scale consists of five different assessments (latching, audible swallowing, nipple type, comfort, and hold) with scores of 0 to 10, indicating poor sucking to normal sucking. Both the sucking rate and the LATCH scores were collected at the first day of intervention and after 2 weeks of intervention as the pre-test and post-test values respectively [21].

**Oro-motor intervention protocol**

- Oro-motor intervention is essential for preterm and term neonates with sucking difficulty.
- Criteria: Neonates are enrolled if they produce less than 10 sucks per minute.
- Before starting the procedure assess for rooting reflex. If neonate doesn’t initiate rooting then therapy begins with initiation of rooting followed by perioral and intraoral stimulation.
- Precautionary measures before Oro-motor intervention: Monitor vital signs (Temperature, SpO_2_, Pulse Rate, Heart rate, Blood Pressure). Any external appliances (Ventilator, OG tube, Oxygen mask, Incubator). Any Congenital Facial Anomalies.

## Statistical analysis

The Means and standard deviations of the collected data were calculated at pre-test and post-test of all the 6 samples, sucking rate (8.66 +/– 3.24), (32.5 +/– 10.46) and LATCH scores (4.66 +/– 0.47), (8.16 +/– 1.46). The values were statistically analyzed using paired t-test to determine the null hypothesis and the data analysis will be considered significant if the p value < 0.05.

## Results

Both term and preterm neonates enrolled in this study with the male female ratio of 4:2. Irrespective of their birth weight neonates with sucking difficulty and on nasogastric tube feed are enrolled in this study. The study was aimed to determine the effect of Oro-motor intervention protocol on immature sucking behavior. Sucking rate counted for continuous 3 minutes and average value of 2nd minute was taken in this study and LATCH score of Pre-test was tabulated in ***Table 1***. After 2 weeks of intervention the post-test value of sucking rate and LATCH score were collected and tabulated in ***Table 2***.

**Table 1:** Intervention protocol.


STIMULTION	PROCEDURE

Perioral	Buccinators muscle massage: Massage given in anticlockwise and clockwise direction [19,28]. (1 min) Compression: Given from ear to corner of the mouth in both right and left cheeks (1 min), upper lip from base to the nose to corner of the lips and lower lip from base of the chin to corner of the lower lip (1 min) [19,30]. Duration: 3 minutes.

Intraoral	Mild stroking done on the hard palate (1 min), side of the gums and then on the tongue with little finger (1 min) [19]. Duration: 2 minutes


**Table 2:** Baseline criteria of 6 subjects.


SUBJECT	GESTATIONAL AGE	GENDER	BIRTH WEIGHT	TUBE FEEDING	SUCKING RATE/MINUTE	LATCH

1	35 weeks	Male	2.460	NG tube	8	5

2	34 weeks + 4 days	Female	2.00	NG tube	9	4

3	31 weeks + 3 days	Male	1.600	NG tube	14	5

4	38 weeks	Male	3.100	NG tube	4	4

5	36 weeks + 4 days	Female	2.150	NG tube	11	5

6	36 weeks + 4 days	Male	2.520	NG tube	6	5


The mean pre-test and post-test values of sucking rate were (8.66), (32.5) and LATCH were (4.66), (8.16) respectively. The table t value was calculated using paired t-test and represented in ***Table 3***. The calculated t value of sucking rate was 6.96, at p value < 0.05, similarly the calculated t-value of LATCH score was 6.216 at p value < 0.05. Thus, both the results were statistically significant indicating the sucking rate and LATCH scores were improved after 2 weeks of intervention among infants with immature sucking behavior (***Table 4***).

**Table 3:** Post-test value of sucking rate and LATCH score.


SUBJECT	GENDER	SUCKING RATE/MINUTE	LATCH

1	M	40	10

2	F	33	8

3	M	47	10

4	M	15	6

5	F	36	8

6	M	24	7


**Table 4:** Pre-test and post-test comparison.


S.NO	OUTCOME MEASURES	PRE-TEST MEAN +/– STANDARD DEVIATION	POST-TEST MEAN +/– STANDARD DEVIATION	T-VALUE	P-VALUE

1	Sucking rate	8.66 +/– 3.24	32.5 +/– 10.46	6.96	0.0009

2	LATCH score	4.66 +/– 0.47	8.16 +/– 1.46	6.216	0.0016


From the statistical analysis the data collected showed that the protocol framed for Oro-motor intervention was significantly effective in improving quality of feeding in infants with immature sucking behavior.

## Discussion

Various treatment methods were previously adopted to improve the sucking pattern such as music, auditory, vestibular and tactile stimulation and stroking to improve non-nutritive sucking [17,18,19], but there is still a lag in the structured protocol for improving sucking behavior in infants with immature sucking. Thus, the purpose of this study is to develop a structured protocol for the Oro-motor intervention to improve sucking behavior.

Advanced treatment techniques were also have been available but still they need an experienced person for clinical practice [22,23,24,25,26,27]. Medoff-Cooper B et al, used multisensory approach to improve sucking pattern such as auditory, tactile and vestibular stimulation to improve feeding performance in infants [18]. Similar to our study, Fucile et al, used peri-oral and intra-oral stimulation to improve feeding in preterm infants, which showed significant improvements in overall milk intake, rate at which the milk transfer that is milliliter per minute and amplitude of expression in mmHg, thus the methods used in the structured protocol may also be effective in improving milk intake rate and milk transfer [28].

Gaebler CP et al, used stroking and perioral and intraoral stimulation as pre feeding protocol but there is a lag in exact duration in which the procedure to be followed [19]. Infants with sucking difficulty have a risk of oropharyngeal milk aspiration. Early intervention aimed to improve the sucking behavior in such infants [29]. To prevent aspiration some neonates have been in nasogastric tube in NICU, future they might have risk of infection spread [15,16]. Neonates without initiation of sucking reflex or delay in sucking behavior may develop speech difficulty [6].

Because of the methodological limitations, only 6 infants were included in the study. This might have resulted in a weak statistic (i.e., a small sample size). However, the current study suggests that the intervention may in fact be improving the sucking behavior, the small sample size in the study was considered as the major limitation. This raises queries whether significant statistical difference could be obtained in a larger sample size. Another possibility is that the outcomes used in this study were not sufficiently sensitive to adequately measure the intervention effect on sucking behavior. The use of more sensitive instruments such as real-time observation techniques, and sucking apparatus would be helpful in clarifying the results.

Oro-motor intervention protocol begins with monitoring the vitals followed by initiation of rooting reflex, perioral and intraoral stimulation. Subjects in this study presented with sucking difficulty during direct breast-feed, due to prematurity sucking reflex is not completely matured, and because of post natal complications such as encephalopathy, seizure disorder and respiratory distress syndrome, neonates have difficulty in maintaining rhythmic pattern of suck-swallow-breath during direct breast feeding.

## Conclusion

The current study analyzed the effectiveness of the structured Oro-motor intervention protocol and shows improvement in feeding performance of infants with poor sucking behavior and improves the LATCH score. All the infants included in this study where under nasogastric tube feeding, thus the structured protocol can be considered to be helpful in weaning from NG tube feeding. However, further studies with large sample size and randomization are needed to determine the absolute effect of the current Oro-motor intervention protocol.
